# MALDI-TOF as a powerful tool for identifying and differentiating closely related microorganisms: the strange case of three reference strains of *Paenibacillus polymyxa*

**DOI:** 10.1038/s41598-023-50010-w

**Published:** 2024-01-31

**Authors:** Ilaria Lebano, Fabio Fracchetti, Mario Li Vigni, Juan Fernando Mejia, Giovanna Felis, Silvia Lampis

**Affiliations:** 1 Syngenta Biologicals (Valagro SpA), 66041 Atessa, CH Italy; 2 Microbion S.r.l., 37057 San Giovanni Lupatoto, VR Italy; 3 Department of Biotechnology and VUCC-DBT Verona University Culture Collection, University of Verona, 37154 Verona, VR Italy

**Keywords:** Biotechnology, Microbiology

## Abstract

Accurate identification and typing of microbes are crucial steps in gaining an awareness of the biological heterogeneity and reliability of microbial material within any proprietary or public collection. *Paenibacillus polymyxa* is a bacterial species of great agricultural and industrial importance due to its plant growth-promoting activities and production of several relevant secondary metabolites. In recent years, matrix-assisted laser desorption ionisation time-of-flight mass spectrometry (MALDI-TOF MS) has been widely used as an alternative rapid tool for identifying, typing, and differentiating closely related strains. In this study, we investigated the diversity of three *P. polymyxa* strains. The mass spectra of ATCC 842^T^, DSM 292, and DSM 365 were obtained, analysed, and compared to select discriminant peaks using ClinProTools software and generate classification models. MALDI-TOF MS analysis showed inconsistent results in identifying DSM 292 and DSM 365 as belonging to *P. polimixa* species, and comparative analysis of mass spectra revealed the presence of highly discriminatory biomarkers among the three strains. 16S rRNA sequencing and Average Nucleotide Identity (ANI) confirmed the discrepancies found in the proteomic analysis. The case study presented here suggests the enormous potential of the proteomic-based approach, combined with statistical tools, to predict and explore differences between closely related strains in large microbial datasets.

## Introduction

*Paenibacillus* is a ubiquitous genus consisting of 280 bacterial species able to colonise different environmental niches, having great relevance in the industrial and agricultural fields^1,2^.

*Paenibacillus polymyxa* is the type species of the genus *Paenibacillus*^3,4^. Members of such species are undoubtedly the most studied and characterised because of their beneficial properties and ability to produce an extensive repertoire of industrially relevant secondary metabolites^5,6^. Indeed, many *Paenibacillus polymyxa* have been reviewed for their plant growth promotion activity through phosphorus solubilization^2^, nitrogen fixation^7,8^, siderophores, phytohormones production, and organic matter degradation^9^.

Among the secondary metabolites of *P. polymixa* with biotechnological and medical applicability, there are antimicrobial compounds (e.g., polymyxins and fusaricidins)^10^, exo-polysaccharides (EPS)^11^, hydrolytic enzymes (e.g., amylases, pectinases, cellulases, hemicellulases)^5,10^ and 2,3- Butanediol (2,3-BDO). This last valuable compound has a vast range of applications in the chemical, pharmaceutical, and food industries as a precursor or additive of many manufacturing processes^12–14^.

One of the most efficient producers of 2,3-BDO optically active isomers is *Paenibacillus polymyxa* DSM 365^15,16^, a generally recognised safe (GRAS) microorganism. This strain was isolated from garden soil^17^, and the genome was sequenced completely^16^. Genome annotation disclosed several traits related to biostimulant activity (e.g., nitrogen fixation, siderophores and EPS biosynthesis)^16^. Moreover, the strain was also described as a biocontrol agent able to induce the Immune Systemic Resistance (ISR) of tobacco plants against the pathogen *Phytophthora parasitica*^18^.

Besides *P. polymyxa* DSM 365, another *P. polymyxa* strain having relevant biotechnological potential is DSM 292, a platform for heterologous protein production^19^. Indeed, the low proteolytic activity would make it an ideal host for the high-yield and stable production of enzymes^19,20^.

Since DSM 365 and DSM 292 are microbial cultures available in public collections and present relevant biotechnological and industrial applications, a thorough investigation of their biodiversity is particularly interesting.

Matrix-assisted laser/desorption ionisation time-of-flight mass spectrometry (MALDI-TOF MS) is a technology that has widely been introduced in many laboratories as a high-throughput, cost-effective, fast, and reliable tool to identify, classify and typing unknown microorganisms. It allows obtaining a mass fingerprint profile for each unknown strain that is matched against the reference spectra in the instrument database to assign an identity.

In this study, we tested MALDI-TOF MS analysis integrated with statistical tools to explore the biodiversity among DSM 365, DSM 292, and the type strain of the species ATCC 842^T^. In addition, 16S rRNA sequence analysis and Average Nucleotide Identity (ANI) were performed to assess and verify MALDI-TOF MS results. Phenotypical test and fatty acids cellular composition analysis were also performed to assess putative differences among the three strains here taken into consideration. This work represents, in our opinion, an interesting case study that highlights the potential of the proposed complementary typing approach to improve the identification resolution of very closed related strains in any proprietary or public culture collection, making it possible to enrich any *in-house* reference database. This approach to biodiversity screening could precede genomic analyses as a kind of predictive tool, able to point to interesting discrepancies that need to be investigated further with a targeted, in-depth approach.

## Results

### MALDI-TOF MS identification results

In this study, the identification of ATCC 842^T^, DSM 292, and DSM 365 with the BDAL library through the MBT Compass (Bruker Daltonik GmbH, Bremen, Germany) software did not provide the expected results. More specifically, the identification of DSM 292 and DSM 365 did not reach the minimum log(score) for a high match with the reference spectra in the database; thus for 8 out of 15 of DSM 292 and 14 out of 15 of DSM 365 signal replicates the identification was confirmed only at the genus level, with a log(score) ranging from 1.710–1.840 and 1.720–1.990, respectively. Conversely, regarding ATCC 842^T^, 13 out of 15 signal replicates were correctly identified as *P. polymyxa* DSM 36^T^ (reference spectrum DSM 36T DSM_2) with a log(score) ranging from 2.010 to 2.250 (Table S1). Furthermore, it was noticed that both the profiles of the DSM 292 and the DSM 365 were even more different from the reference spectrum of the type strain DSM 36 T since, for most replicates, it was not reported among the closest classification results, and the matching resulted in not reliable identification. Indeed the identification (log)score for DSM 365 and DSM 292 compared to DSM 36 T DSM_2 ranged from 1.520–1.920 and 1.050–1.850 (Table S2).

**Figure 1:**
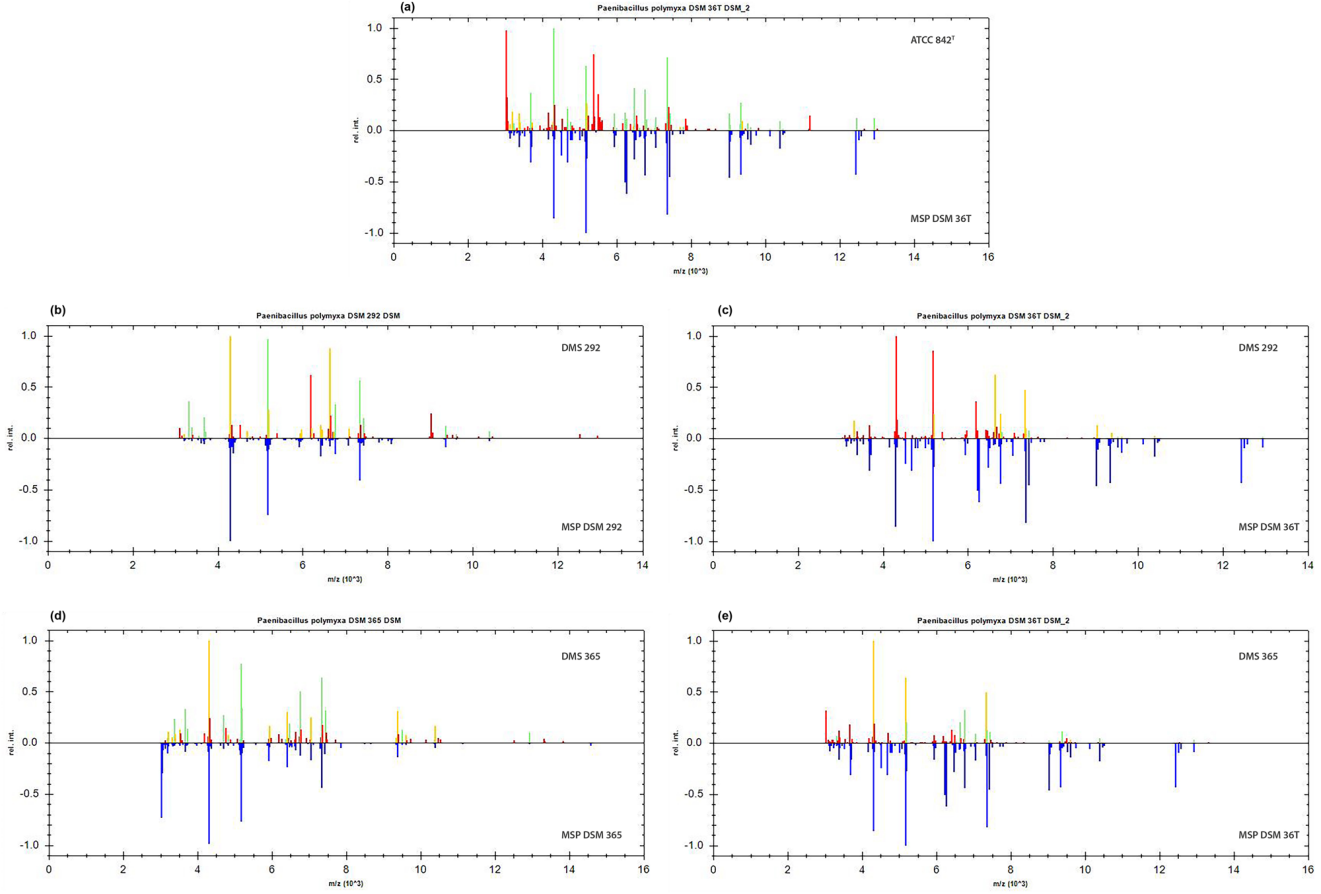
Graphical representation of *P. polymyxa* identification by MALDI Biotyper Software. The colour of the peaks reflects the degree of matching of (**a**) ATCC 842^T^, (**b**,**c**) DSM 292, and (**d**,**e**) DSM 365 with the reference MSPs (green = full match, yellow = partial match and red = mismatched) reported in the lower part of the graphs (blue spectra).

The matching degree between each analysed strain and the reference Main Spectrum Profile (MSP) is reported in Fig. 1.

### Mass data statistical analysis results and *in-house* database implementation with DSM 292 and DSM 365 profiles

**Figure 2:**
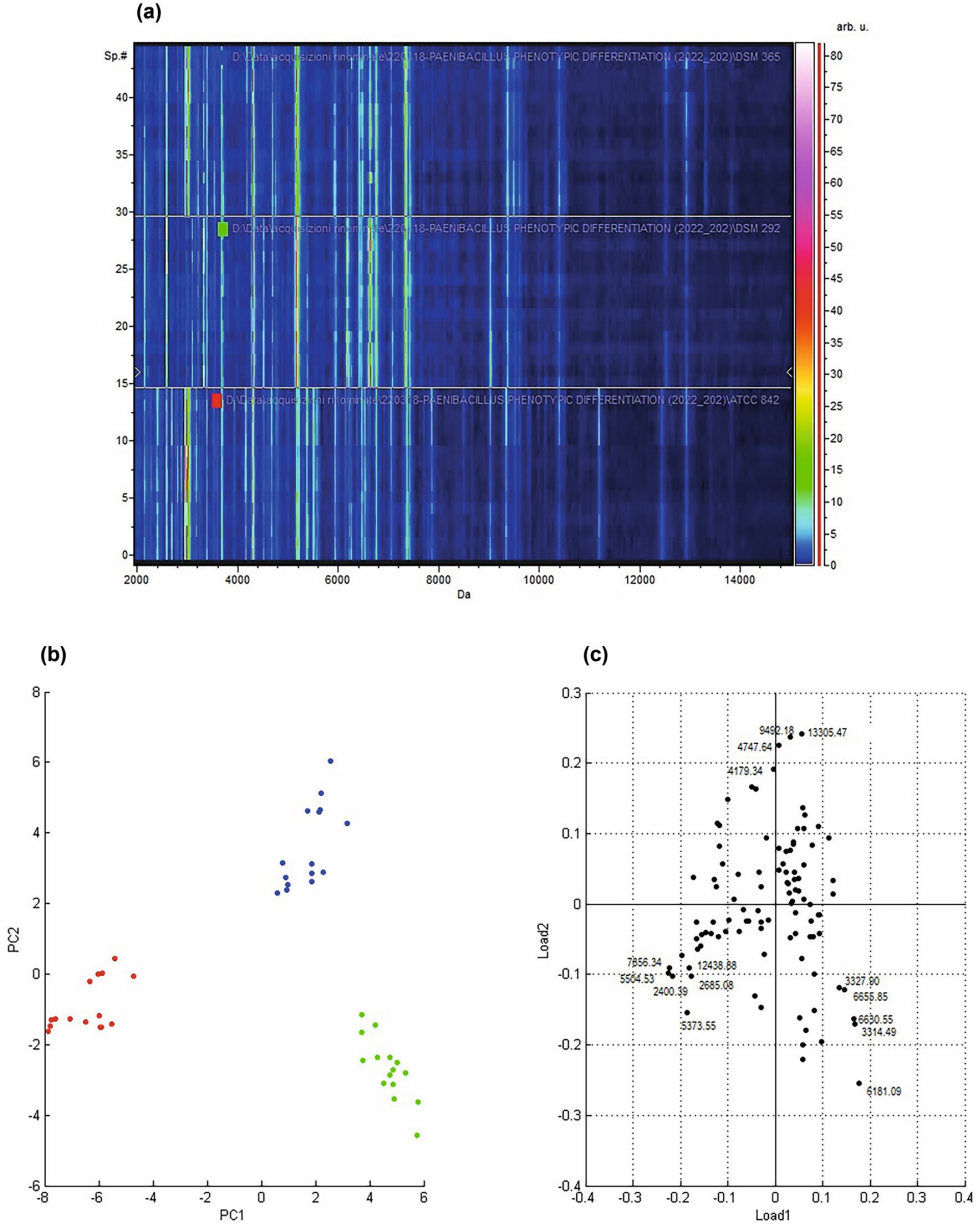
Mass spectra from 2000 to 15,000 Da of ATCC 842^T^, DSM 292 and DSM 365 were processed and analysed by ClinProTools software. (**a**) Pseudo-gel like view of ATCC 842^T^ (red), DSM 292 (green) and DSM 365 (blue) mass spectra (15 replicates per class) displayed on a rainbow scale. Pseudo-gel like view of ATCC 842^T^ (red), DSM 292 (green) and DSM 365 (blue) mass spectra (15 replicates per class) displayed on a rainbow scale. The colour bar on the right side of the gel view indicates the peak intensity; 2-D PCA (**b**) and Loadings of 2D-PCA (**c**) plots of ATCC 842^T^ (red), DSM 292 (green) and DSM 365 (blue).

Given the preliminary results obtained in the identification step, the mass spectra of the DSM 292 and 365 were compared with the mass profile of ATCC 842^T^ to assess whether there were consistent differences and discriminative biomarkers in their mass fingerprint that could explain the mismatch with the type strain. All the 15 raw mass spectra for each strain were loaded into the ClinProTools software (v 3.0; Bruker Daltonics, Bremen, Germany) and processed following the standard data preparation workflow (baseline subtraction, normalisation, recalibration, average peak list calculation and peak calculation)^1^. A pseudo-gel-like view of the processed spectra loaded per class is reported in Fig. 2a. Moreover, the average spectra view of all three classes is reported in Figure S3. As a first step, Principal Component Analysis (PCA) was performed to get more information about the data set variability and clustering of the three *Paenibacillus* strains. The two-dimensional representation of the scores (PC1 vs. PC2, Fig. 2b and Figure S2) showed that the three *P. polymyxa* strains grouped differently. The loadings plot (PC1 vs. PC2, Fig. 2c and Figure S2) offers a visualisation of the peaks that influence this clustering the most, indicating that the three strains showed spectral differences in their mass fingerprint. Moreover, the study suggested that the peaks influencing this clustering could be potential discriminating markers.

**Figure 3:**
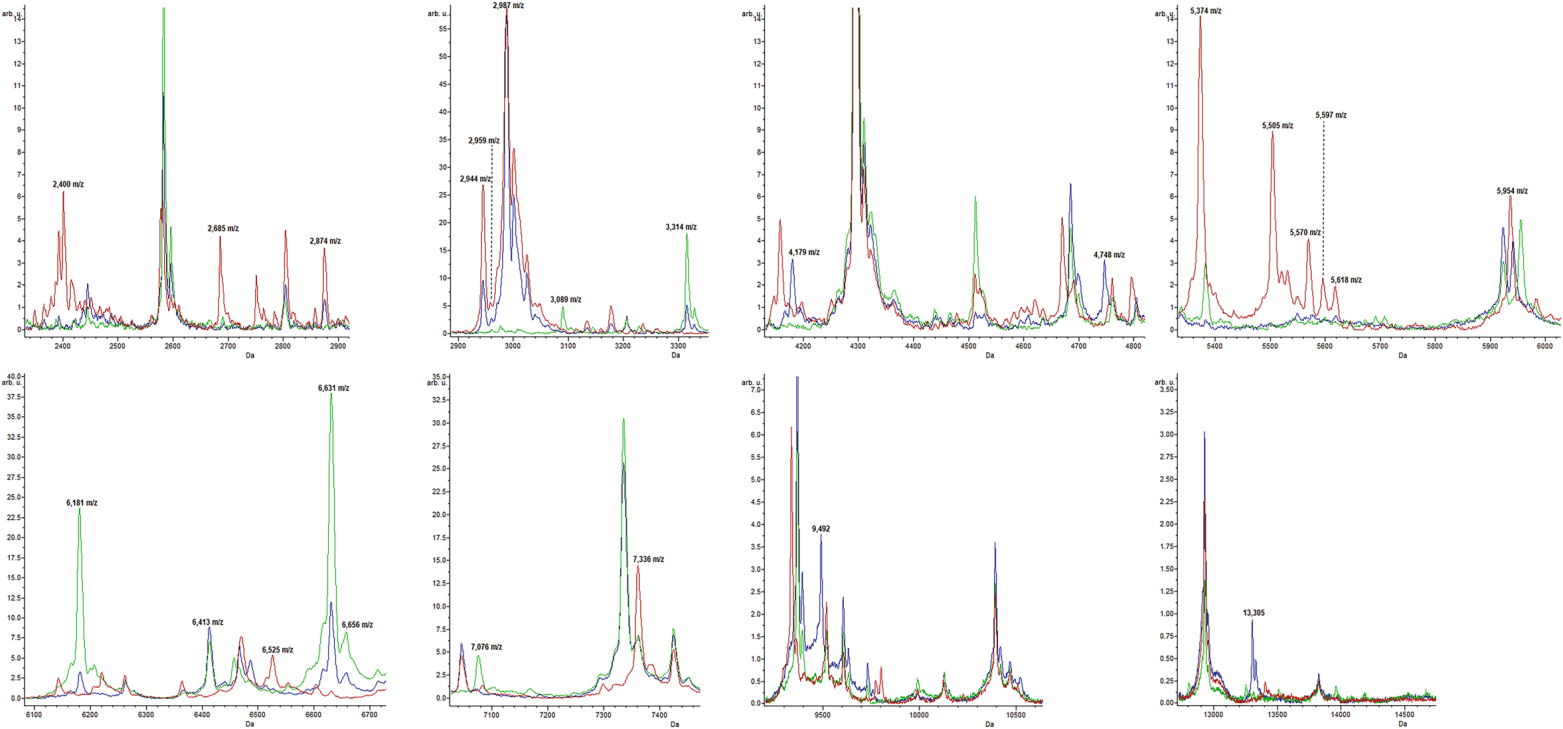
Average spectra of the most characteristic peaks among *P. polymyxa* strains. Intensities of characteristic peaks in ATCC 842^T^ (red), DSM 292 (green) and DSM 365 (blue) are shown on y-axes and expressed in arbitrary intensity units.

Subsequently, after the visual comparison of *P.polymyxa* mass spectra, the selection of peaks showing a significant difference in the average intensity among ATCC 842^T^, DSM 292 and DSM 365 was made according to the *p*-values (≤ 0.05) obtained through Wilcoxon/Kruskal Wallis (W/KW) analysis since the *p*-value for AD (Anderson–Darling) test was less than equal to 0.05 (Table 1)**.** The selected masses were confirmed to be very informative in discriminating the three *P. polymyxa* strains since the *p*-value was < 0.000001 for all. Moreover, such biomarkers strongly affected the clustering in the PCA (Fig. 2c). Peaks describing the fingerprint of each strain the most were identified by measuring their change in intensity average (log_2_ fold change) as reported in Table 1. Results showed that high expression levels of peaks *m/z* 2400, 2685, 6525, and the region from *m/z* 5505 to 5954 strongly described the fingerprint of ATCC 842^T^. Similarly, low-intensity levels of *m/z* 6413 and 7336 were good descriptors of such a profile. Low intensities of *m/z* 2959 and 2987 and high intensities of *m/z* 3089 and 7076 described the DSM 292 profile well. Furthermore, high expression levels of *m/z* 4179, 4748, 9492 and 13,305 were indicative of the profile of DSM 365. Some discriminant characteristics of the selection (*m/z* 2874, 2944, 3314, 5374, 6181, 6631, 6656) proved to be good descriptors of all three classes simultaneously, as their intensity values were quite distinct. The average intensities of the above mentioned peaks are reported in Fig. 3.

**Table 1:**
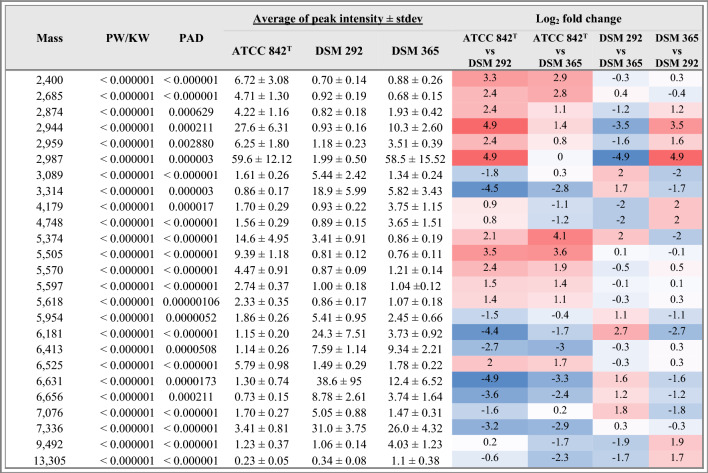
List of characteristic mass peaks selected according to the *p*-value (≤ 0.05) obtained through W/KW analysis.

Changes in peak average intensity between classes are reported as a heatmap of log_2_ fold change.

**Figure 4:**
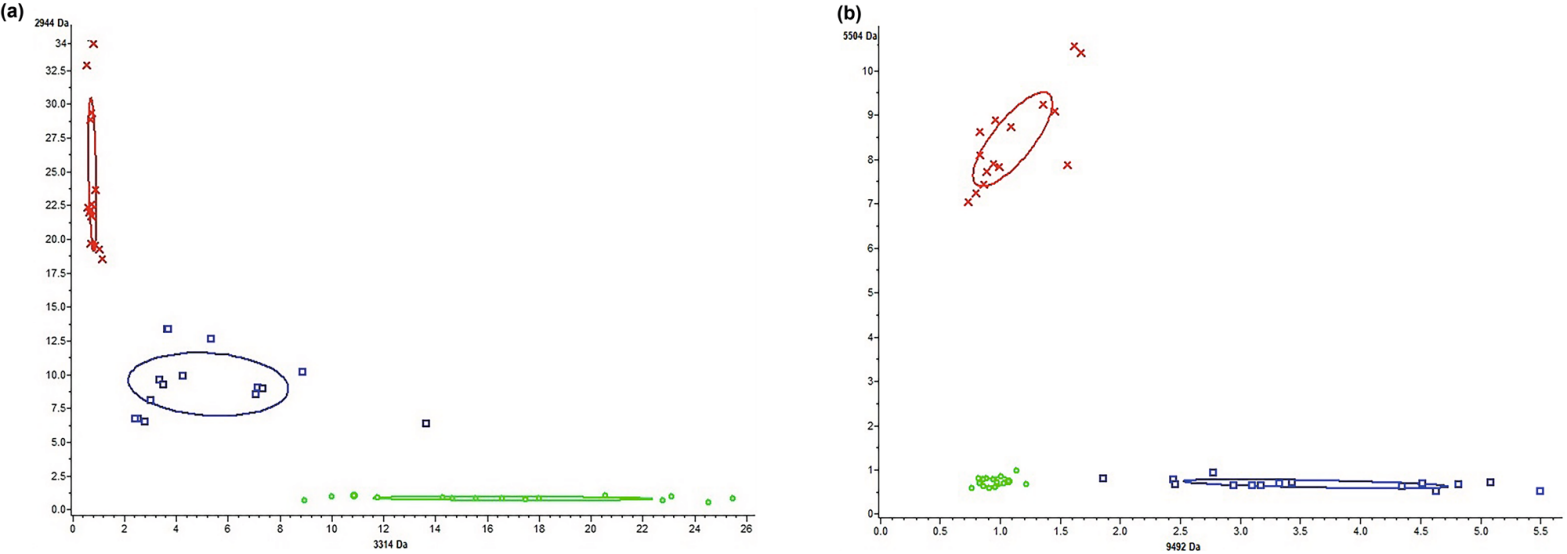
2D scatter plots of characteristic peaks for ATCC 842^T^ (red), DSM 292 (green) and DSM 365 (blue) selected by QC and SNN classification models. (**a**) Scatter plots of peaks 2944 and 3314 Da (selected by the QC model) and (**b**) 5504 and 9492 Da (selected by the SNN model). The intensities of peaks were expressed in arbitrary intensity units and served as y-axes. The ellipses represent the 95% confidence intervals of peak intensity for each strain.

Classification models (CMs) were generated by ClinProTools software using Quick Classifier (QC)^21^, Supervised Neural Network (SNN)^21^ and Genetic Algorithm (GA)^21,22^ to obtain systematic and objective strain discrimination according to a defined list of discriminative biomarkers selected by the algorithm. The performance of all classification models was calculated to choose the best able to discriminate the three *P. polymyxa* strains simultaneously. According to the results reported in Table 2, GA_KNN algorithms with 1, 3 and 5 nearest neighbor settings showed very high discriminating power (100% accuracy) for ATCC 842^T^ and DSM 365 classes but not for DSM 292. The only multiclass models able to correctly assign and classify an independent data set into one out of three classes were SNN and QC, with a cross-validation value of 100%. Informative features selected by such multiclass classification models are reported in Table 3. Such peaks showed different expression patterns and high discriminatory power, as shown in 2D scatter plots reporting strain distributions according to peak intensity (Fig. 4).

**Table 2:** Performance of classification models of ATCC 842^T^, DSM 292 and DSM 365.

Strain	Model	Selected peaks	Accuracy (%)	Sensitivity (%)	Specificity (%)
ATCC 842^T^	GA_KNN1	30	100	100	100
	GA_KNN3	8	100	100	100
	GA_KNN5	5	100	100	100
	GA_KNN7	4	45.90	38.10	50
	SNN	3	100	100	100
	QC	4	100	100	100
DSM 292	GA_KNN1	30	90.16	70	100
	GA_KNN3	8	98.36	95	100
	GA_KNN5	5	95.08	85	100
	GA_KNN7	4	58.34	0	100
	SNN	3	100	100	100
	QC	4	100	100	100
DSM 365	GA_KNN1	30	100	100	100
	GA_KNN3	8	100	100	100
	GA_KNN5	5	100	100	100
	GA_KNN7	4	100	100	100
	SNN	3	100	100	100
	QC	4	100	100	100

Accuracy, sensitivity and specificity are reported as a percentage. Each of the parameters was calculated as follows: Accuracy = TP + TN / TP + TN + FP + FN; Sensitivity = TP/ TP + FN; Specificity = TN/ TN + FP. TP, true positive; FN, false negatives; TN, true negatives; FP, false positives (FP).

**Table 3:** List of discriminative peaks according to QC and SNN classification models.

CMs	Mass	PW/KW	PAD	Average of peak intensity ± stdev		
				ATCC 842^T^	DSM 292	DSM 365
QC	2944	< 0.000001	0.000211	27.55 ± 6.31	0.93 ± 0.16	10.34 ± 2.6
	2959	< 0.000001	0.002700	8.08 ± 2.35	1.52 ± 0.29	4.50 ± 0.50
	2874	< 0.000001	0.000528	5.45 ± 1.52	1.05 ± 0.22	2.47 ± 0.53
	3314	< 0.000001	0.0000035	0.86 ± 0.17	18.87 ± 5.99	5.82 ± 3.43
SNN	5505	< 0.000001	< 0.000001	9.39 ± 1.18	0.81 ± 0.12	0.76 ± 0.11
	7076	< 0.000001	< 0.000001	2.19 ± 0.35	6.48 ± 1.13	1.89 ± 0.41
	9492	< 0.000001	< 0.000001	1.23 ± 0.37	1.06 ± 0.14	4.03 ± 1.23

Given the successful performance of multiclass models in classifying and recognising the three strains of *P. polymyxa*, the independent signal sets tested in cross-validation were used to generate new MSPs for DSM 292 and DSM 365 inside the *in-house* reference library.

After implementing the library with the newly generated MSPs for DSM 292 and DSM 365, the identification results of the fifteen spectra showed improvement. These results exhibited a higher agreement in terms of log(score) with the internally generated MSPs compared to the reference profiles in the MBT Compass library (Table S3).

### 16S rRNA sequence retrieving from genomes and comparison

The identification of the strain ATCC 842^T^ (= DSM 36^T^), DSM 292, and DSM 365 by comparison of the assembled Sanger sequence in EzBioCloud confirmed, as expected, the identification of the strain ATCC 842^T^ as *P. polymyxa*. Contrarily the best hit resulting after the analysis of the 16S rRNA sequence of the strains DSM 292 (1372 bp) and DSM 365 (1385 bp) was *Paenibacillus peoriae*, with respectively 99.78% and 99.85%) in the case of the sequence from the strain DSM 365 the second-best hit was *Paenibacillus ottowi* (99.56%), both the species belonging to the *P. polymyxa* group. The species *P. polymixa* is, therefore, respectively, the second and third-best hit with a % similarity of 99.42 and 99.27. Remarkably, when the Sanger sequence obtained from the strain DSM 292 is compared with the most similar 16S rRNA locus sequence retrieved from the genome (OXKC02000021.1), the similarity percentage is 99.93%. Analogously, the comparison of the sequence of the most similar 16S rRNA locus sequence retrieved from the genome of the strain DSM 365 (JAKVDC010000038.1, contig 38) showed 99.92% similarity.

### Genome comparison results

The relatedness of the strains by means of a whole-genome approach was defined by calculating the ANI with the OrthoANI algorithm and setting 95–96% as the threshold to propose new species^23^. The ANI values of the strain DSM 365 and DSM 292 clearly separate these two strains from ATCC 842^T^, the type strain of *P. polymyxa* species, as they resulted in 90.12 and 89.99, respectively. Based on this result, the strains DSM 365 and DSM 292 are distinguishable from the type strains, and they can be suggested as references of a cluster of *P. polymyxa* strains different from the *P. polymyxa *sensu stricto. Remarkably, the ANI value resulting from the comparison of the strains DSM 365 and DSM 292 was close to the threshold, i.e., 95.40; therefore, it was not possible to clearly support the presence of a third emerging cluster nucleus within the *P. polymyxa* species.

The presence of remarkable genes and/or biosynthetic pathways were searched by BLASTn in the three previously reported genomes in order to enlighten differences in the presence of Butanediol Dehydrogenase (GenBank Acc. No.: JMIQ01000019: 17,969..19021)14 and Polymyxin Synthetase (EU371992.1:22,102-41,040)38.

Considering peculiar genes and biosynthetic pathways, it was possible to evidence that the strain DSM 365 is the only strain among the three compared here to harbour the Butanediol Dehydrogenase gene. A genetic locus with different nucleotide similarity was found in both the other two strains: the most related gene in the strain DSM 292 was annotated as Sorbitol Dehydrogenase (GenBank Acc. No. OXKC02000003.1:428,721–429,773, locus_tag “PPOLYM_01857”,), with only 96.39% nucleotide similarity. In the genome of the strain ATCC 842^T^, which is not annotated, it was not possible to find any predicted function, but a 100% coverage region with only 94.11% nucleotide similarity was found, suggesting that region (CAIGJZ010000013.1:3117-4169) does not code for Butanediol Dehydrogenase gene.

Concerning Polymyxin Synthetase, it was not possible to find the complete query in only one contig of the three genome strains, suggesting it was not completely assembled. More *in-silico* or *in-vivo* analyses are required to evaluate this genetic function.

### Phenotypical test (API) and fatty acids cellular composition results

Comparison results about substrate utilisation revealed that the strains differed for some traits (Table 4). In particular, both *P. polymyxa* DSM 292 and DSM 365 showed different behaviour in acetylmethylcarbinol production^24^ according to the Voges-Proskauer (VP) assay compared to the type strain *P. polymyxa* ATCC 842^T^. Additionally, *P. polymyxa* DSM 292 was positive in malate assimilation, rhamnose, and α-methyl-d-mannoside utilisation and weak in tween 80 degradation compared to ATCC 842^T^ and DSM 365. Further differences can also be observed in growth temperature and tolerance to 5% of NaCl. More specifically, DSM 365 grew at higher temperatures (35 °C), and DSM 292 resulted in being not tolerant of 5% of NaCl.

**Table 4:** Comparison of main differing features in the phenotypical characterisation and fatty acids cellular composition of ATCC 842^T^, DSM 292 and DSM 365.

Kit name	Feature	ATCC 842^ T^	DSM 292	DSM 365
	5% NaCl	W	–	W
	35 °C	W	W	+
	TWEEN 80	W	–	+
API 20 E	Voges-Proskauer (VP)	W	+	+
API 50 CHB	Rhamnose	–	+	–
API 50 CHB	α-methyl-d-mannoside	–	+	–
API 20 NE	Malate assimilation	–	+	–
API ZYM	Alcaline Phosphatase	0.5	0.5	1
API ZYM	Leucine-Arylamidase	0	1	0
API ZYM	Chymotrypsin	0.5	0	0
API ZYM	Acid Phosphatase	0.5	0	0
API ZYM	Naphthol-AS-BI-Phosphohydrolase	0.5	1	0.5
API ZYM	α-Galactosidase	1	1	2
API ZYM	β-Galactosidase	3	4	5
API ZYM	β-Glucosidase	0	0.5	0.5

Fatty acid	ATCC 842^T^	DSM 365	DSM 292	
15:0 anteiso	46.7	61.3	61.6	
16:00	11.5	6.3	6.9	
15:0 iso	8.6	6.9	7.1	
17:0 anteiso	8.3	7.2	9	
16:0 iso	8.1	7	5.3	
17:0 iso	5.5	2.9	3.8	
16:1 w11c	4.1	2.4	0.9	
14:00	2.1	1.4	1.4	
14:0 iso	1.7	1.3	1	
15:00	0.8	1.8	1.9	
17:1 iso w11c	0.6	0.4	0.2	
16:1 ISO w10c	0.5	0.5	ND	
17:00	0.4	0.2	0.3	
17:1 w6c	0.3	ND	0.7	

Regarding the production of enzymes, colourimetric reactions of different intensities were recorded in the API ZYM kit. Differences in colour reactions might reveal potential differences in the enzyme production profiles of the three strains. Among the positive recordings, DSM 365 showed the highest intensity reaction for β-Galactosidase (Table 4 and Table S8). The entire list of substrate utilisation and details about morphological description (including cell pictures) can be found in Supplementary Figure S1, Table S4, Table S5, Table S6, and Table S7 online.

The comparison of fatty acids profiles (Table 4) revealed that all three strains have 15:0 anteiso, 16:00, 15:00 iso, 17:0 anteiso, 16:0 iso and 17:0 iso as major cellular fatty acids. Moreover, the fatty acid 16:1 w11c was the one that differed the most among the three strains.

## Discussion

MALDI-TOF MS has been widely introduced and applied in the last few years as an identification technique and an alternative rapid approach for typing and discriminating microbial strains at the subspecies level^25–27^. Such a technology allows obtaining a mass fingerprint profile for the analysed strain, enriched in highly abundant ribosomal proteins, which is compared with reference protein spectra contained in a database to assign an identification result.

Although ribosomal proteins are strongly conserved in the bacterial species, they may present modest variation at the microbial strain level^28^. Several authors proposed using the MALDI-TOF MS typing method coupled with statistical tools to classify and improve the identification and differentiation of phylogenetically closely related species^27,29–31^. Indeed, adopting such an approach allows for identifying a certain number of discriminative and reproducible biomarkers with a specificity at the subspecies level^32^.

In this study, we exploited the potential of unsupervised classification methods coupled with proteomic fingerprinting analysis to explore the diversity of three strains belonging to *P. polymyxa*. *P. polymyxa* is one of the most extensively described and discussed species due to its wide applicability in the biotechnological and agricultural fields^2^. This is evidenced by an increasing number of patents and genome sequencing projects in recent years. A search for patents using the genus *Paenibacillus* or the species *polymyxa* as query words returns 6428 and 2477 results, respectively (https://worldwide.espacenet.com). To date, the number of *P. polymyxa* genome sequencing projects available on NCBI and the Genome Taxonomy Database (GTDB) are 93 and 95 (https://gtdb.ecogenomic.org/), respectively. The increasing number of genome sequencing projects may reflect the need to retrieve functional information from the genetic code and even solve the intrinsic taxonomic complexity of the *Paenibacillus* genus and *polymyxa* species^2,33^.

In addition, several works based on MALDI-TOF MS analysis have interested the genus *Paenibacillus* in recent years. Most of them focused on the identification, detection and typing of strains associated with pathogenicity potential and food spoilage^34,35^, production of antimicrobial compounds^36^ and interaction with the plant^37^.

The number and quality of reference spectra within the instrument database can greatly influence the success rate of identification using the proteomic approach^38^. The current Bruker Daltonics database contains 251 MSPs of *Paenibacillus* and ten profiles of *P. polymyxa.* These include the reference spectra of *P. polymyxa* ATCC 842^T^, DSM 365 and DSM 292.

In this study, the preliminary identification of the purchased *P. polymyxa* cultures with the manufacturer's database was inconclusive for *P. polymyxa* DSM 365 and DSM 292. In fact, the matching with their reference spectra did not reach the minimum log score required for a reliable identification at the species level. It is possible that the mass spectra of the two bacterial strains included in the database are still not sufficiently reproducible and should be updated with two new versions. Similarly, the strain ATCC 842^T^ matched best to a more recent version of the DSM 36 T reference spectrum (DSM 36T DSM_2) (Fig. 1). We also noticed marked differences when comparing the profiles of the DSM 365 and the DSM 292 with the reference spectrum of the DSM 36 T type strain in the database. These findings led us to further investigate the observed divergence by comparing their mass fingerprints. Subsequent statistical analyses confirmed the observed differences and revealed different discriminating biomarkers among the three *Paenibacillus polymyxa*. The mass distribution of the *P. polymyxa* strains by PCA clearly showed three distinct clusters, most influenced by different markers whose expression pattern was characteristic of each of the strains. According to the gel view observation (Fig. 2) and the list of discriminatory peaks selected in the W/KW analysis, ATCC 842^T^ showed several biomarkers with higher intensity than DSM 365 and DSM 292 (Table 1). In addition, we noticed that peak m/z 2987 (Table 1, Fig. 3) was consistently expressed in both profiles of ATCC 842^T^ and DSM 365 but was remarkably low in DSM 292. This finding suggested that even very low-intensity signals can act as critical discriminators in mass comparisons.

Some of these peaks (*m/z* 2400, *m/z* 2685, *m/z* 2987; Fig. 3) fall in a variable region of the mass spectrum below *m/z* 3000 that is correlated with nonribosomal peptides, metabolites and lipopeptides production, known antimicrobial compounds produced by several microbial species, including *P. polymyxa*^39–41^. Several authors have investigated such a region in the mass spectra of *P. polymyxa* by MALDI-TOF MS to detect and characterise lipopeptides, antibiotics or volatile compounds^42–44^. Interestingly, peaks *m/z* 2,400 and 2,685 were strongly present in the spectrum of ATCC 842^T^ but almost absent in DSM 292 and DSM 365. These results may reveal a different pattern of secondary metabolite production among the three strains. Based on this assumption, we believe that analysis and comparison focused on the low-mass region (m/z 500–3000) could potentially reveal additional differences, better supporting the description of biodiversity and even strain differentiation^39,45^.

Moreover, considering the nature of our investigation into closely related species classification, it is worth noting that the analysis of the lower m/z range (up to m/z 2000) not only pertains to small peptides, as initially discussed, but also encompasses lipids. While our study primarily employed proteomics and genomics approaches, it is crucial to acknowledge that there exists a third option, namely MALDI lipidomics, which can offer valuable insights into the composition and variations of lipids within the studied strains^46,47^. This additional dimension of analysis could provide further support for the reliable classification of closely related species and enhance the comprehensiveness of our findings.

On the other hand, the presence of discriminant peaks in the region above m/z 3000 supported the consistency of the discriminant biomarkers that emerged from the statistical analysis of *P. polymyxa* mass spectra. Indeed, the core region of the fingerprint profile is less variable and is associated with highly abundant ribosomal subunit proteins, small-acid soluble proteins and conserved protein domains^48,49^. We found several peaks in this region that showed significant differences in the average intensity between the fingerprint profiles of the three *P. polymyxa* strains (Table 1, Fig. 3). For example, the region from *m/z* 5505 to *m/z* 5618 proved to be very informative as it was characterised by high-intensity ATCC 842^T^ spectrum signals.

From the list of informative peaks, we identified *m/z* 2944, *m/z* 2874 and *m/z* 3314 as potentially good descriptors of all classes simultaneously (Table 1, Fig. 3). Our assumption was then confirmed by the peak selection of the QC model (Table 3). On the other hand, we found that the SNN classification model behaved differently by selecting a list of three markers, each of which was highly informative for one of the three classes (Table 3). The reproducibility of the observed discriminative biomarkers and, consequently, the reliability of such classification models was definitively confirmed by the simultaneous discrimination of all three strains observed with the external dataset. According to these results, we believe that both models should be applied to classify and predict the future identification of new *P. polymyxa* strains.

MALDI-TOF MS identification is often considered culture-independent and relies heavily on signals derived from ribosomal proteins, which constitute a substantial portion of the spectra. However, it's important to note that less abundant proteins or signals can also play a crucial role in achieving strain-level differentiation^28,50^. These less abundant signals may not necessarily be of ribosomal origin, and the culture conditions, such as the culture medium, can indeed influence their abundance. In our study, the mass spectra analyzed were obtained by cultivating the three strains in the same growth medium and environment. While this approach was essential for the direct comparison of the strains, we acknowledge that it may introduce certain limitations. The uniform culture conditions might not fully represent the potential variations that could occur in different conditions. To address this aspect, it will be necessary in future research to repeat the discriminative analysis in the presence of different growth media or environmental conditions. This approach will help clarify the origin of the discriminative peaks and confirm which ones are indeed attributable to minor alterations in ribosomal protein genes.

The relationship between the three strains was further investigated using the ANI calculation in order to assess and verify the MALDI-TOF MS results obtained. Currently, comparative analysis of fully sequenced microbial genomes is the most successful tool for exploring and assessing molecular differences between microbial strains^51^. Several papers have reported the use of such an approach to distinguish closely related microorganisms through rigorous workflow procedures, including genome sequencing, contigs assembly, annotation, and typing of the compared sequences^52,53^. Complementing the MALDI-TOF MS data with the genome sequence comparison of the three strains, we realised that the observed differences in their mass fingerprint profiles were also reflected in the genetic differences between the three strains. Indeed, from a genetic point of view, both *P. polymyxa* DSM 365 and DSM 292 are clearly distinguishable from ATCC 842^T^, e.g., considering ANI. Otherwise, there were less remarkable differences in the taxonomic relatedness of DSM 292 and DSM 365, as the ANI value was close to the threshold. Similarly, based on the fold change values, we found that both strains shared closer mean intensity values for most of the more significant markers than the ATCC 842^T^. Indeed, when comparing DSM 292 and DSM 365 with ATCC 842^T^, only four and five peaks respectively had a fold change as log_2_ between 0 and 1 (as absolute values). On the contrary, the comparison between DSM 292 and DSM 365 resulted in nine discriminant peaks having a fold change as log_2_ between 0 and 1 (as absolute values).

We also noted a more remarkable discriminatory power and sensitivity of the MALDI-TOF MS-based system compared to other phenotypic typing approaches used in this work. Analysis of substrate utilisation and comparison of fatty acid profiles did not reveal desirable features for a comprehensive description of intraspecific diversity. Indeed, all strains shared similar morphological and biochemical traits with few exceptions (Table 4, Table S4, Table S5, Table S6, Table S7, Table S8). Regarding substrate utilisation, we found that ATCC 842^T^ and DSM 292, unlike DSM 365, were unable to utilise rhamnose, α-methyl-d-mannoside and malate. In addition, according to the semiquantitative enzymatic analysis, naphthol-AS-BI-Phosphohydrolase and β-Galactosidase activities appeared to be different in all three strains. Although such positive results, these results should be considered as preliminary and should be confirmed by specific quantitative enzymatic assays.

All these results provide new insights into the genomic diversity of these *P. polymyxa* strains. They could set the stage for a more comprehensive genomic study involving a larger number of strains. The classification of *P. polymyxa*, according to the framework of the Genome Taxonomy Database, could help to select the representative strains according to genome phylogeny and intra-specific clustering.

Furthermore, we expect that future investigations on new bacterial isolates by MALDI-TOF MS analysis and classification algorithms can help to enrich *P. polymyxa* clusters, thus improving the richness and identification resolution of the *in-house* database.

In conclusion, the MALDI-TOF MS analysis performed in this study revealed disagreement in the identification assignment of DSM 365 and DSM 292 to the *P. polymyxa* species, along with notable differences in their mass spectra compared to ATCC 842^T^. These disagreements were corroborated by genetic analyses, reinforcing the consistency of the proteomic findings. Although this evidence was obtained for a limited set of strains, we believe that MALDI-TOF MS analysis, coupled with statistical tools, has considerable potential to study and compare large microbial datasets. Genome sequencing and comparison can be challenging, requiring highly skilled personnel and costly when dealing with large microbial data collections^54^. The application of such an approach could precede genomic analyses as a kind of predictive tool, helping to gain a greater awareness of the biodiversity contained in any microbial collection and highlighting interesting discrepancies between closely related strains that need to be investigated further with a targeted, in-depth approach. The predictive potential of this tool would allow time-consuming and costly efforts to be avoided if there are no factual assumptions that justify further comparative investigations.

## Methods

### Strain culture condition

For the present study, three bacterial strains belonging to *Paenibacillus polymyxa* species were studied: ATCC 842^T^, DSM 292 and DSM 365. *Paenibacillus polymyxa* ATCC 842^T^ (= DSM 36^T^; = KCTC 3858^T^ ), the type strain of *P. polymyxa* and family *Paenibacillaceae*, was purchased from the American Type Culture Collection. *P. polymyxa* DSM 292 (= CCM 1609; LMG 6320) and *P. polymyxa* DSM 365 were acquired from Deutsche Sammlung von Mikroorganismen und Zellkulturen (DSMZ), Braunschweig, Germany. All bacterial cultures were recovered from lyophilised vials onto Tryptic Soy Agar (TSA; Sigma Aldrich, United Kingdom) and grew at least for 24 h at 30 °C. Next, single colonies were streaked on fresh TSA plates, incubated at 30 °C for 16 h, and identified by 16S rRNA and MALDI-TOF MS before further investigation studies.

### MALDI-TOF MS analysis: sample preparation and identification

Prior MALDI-TOF MS measurements, bacterial samples were processed according to the manufacturer’s instruction, following the extraction method. Briefly, for each bacterial culture ~ 0.1 mg of cell material was directly transferred from a single colony to 1.5 ml tubes containing 300 μL sterile water. Following that, the bacterial samples were dissolved and then inactivated by the addition of 900 μL of absolute ethanol solution, with thorough mixing.

The bacterial samples were then centrifuged at 15,000 rpm for two minutes, and the obtained pellets were dried at room temperature for one hour and treated with an equal volume (approximately 25 μL) of 70% formic acid (FA) and acetonitrile (ACN) to extract proteins for acquisition of mass spectra.

For the analysis, one μL of supernatant was spotted on the MSP 96 polished steel target plate (3 biological and five technical replicates, totalising 15 spots per strain), air-dried and overlaid with 1 μL of 4HCCA matrix solution (10 mg/ml of alpha-cyano-4-hydroxycinnamic acid dissolved in a solution of 50% ACN and 2.5% trifluoroacetic acid [TFA], Sigma-Aldrich, Milan, Italy) to permit sample ionization^55^.

The mass spectrum profile of each strain was acquired by Bruker Microflex™ LT MALDI-TOF mass spectrometer (Bruker Daltonics, Bremen, Germany) equipped with a 60 Hz nitrogen laser, using FlexControl™ software (v 3.4; Bruker Daltonik GmbH, Bremen, Germany) in a positive linear mode within a mass range from 1960 to 22,000 dalton. According to the manufacturer’s instructions, system calibration was performed using the Bruker Bacterial Test Standard (BTS, Bruker Daltonics, Germany) solution able to cover a mass range of spectra acquisition between 3,6 and 17 kDa. Data processing was performed automatically by MBT Compass 4.1.100.10 software (Bruker Daltonik GmbH, Bremen, Germany), and the mass spectra were matched against the instrument library provided by Bruker Daltonic (MBT compass library v 11.0.0.0). The library included a list of 10,833 bacterial reference spectra, containing 86 reference spectra belonging to the *Paenibacillus* genus and 10 grasping to *P. polymyxa* species. The MSPs of *P. polymyxa* ATCC 842^T^ (= DSM 36 T 2), *P. polymyxa* DSM 365, and *P. polymyxa* DSM 292 were already present as reference spectra inside the MBT compass library.

The assessment of the spectra quality was carried out by FlexAnalysis (v 3.4; Bruker Daltonik GmbH, Bremen, Germany), a software for spectra processing (smoothing, baseline subtraction and intensity normalisation) that allows removing all flatline spectra or those with outlier peaks. Next to the quality check step, the spectra were then analysed with the MBT Compass Explorer 4.100.1 module (Bruker Daltonik GmbH, Bremen, Germany) to confirm the identification results obtained with automatic data processing and to observe the graphical representation of the match between the analysed strain and the reference MSPs. The software assigned identification results according to the (log)score value resulting from the matching degree of the unknown spectrum with the MSPs of the Bruker taxonomy. According to manufacturer interpretation, (log)score values between 2.00 and 3.00 and 1.70–1.99 indicated a high- and low- confidence identification, respectively^56^. Lower (log)score values meant that no microorganism identification was possible^56^.

### Statistical analysis of mass spectra

The fifteen spectra of ATCC 842^T^, DSM 292, and DSM 365 were loaded into the Clinprotools software (v 3.0; Bruker Daltonics, Bremen, Germany) to visualise strain-level variations and select discriminant biomarkers among the analysed classes^31,57^. The raw spectra of ATCC 842^ T^, DSM 292, and DSM 365 were fed into the software as three distinct subsets of mass data. Before loading the mass data, spectra preparation parameters were set as follows: resolution = 800; baseline subtraction by top hat baseline method; mass range (m/z) for analysis from 2,000 to 15,000; noise threshold = 2, recalibration = 1,000 ppm for maximal peak shift and 30% match to calibrant peaks. Moreover, peak calculation settings were adjusted as follows: peak picking on the total average spectrum with a signal-to-noise threshold equal to 5.

Next, the following steps were performed for mass spectra preparation of all three classes: recalibration, average peak list calculation, and peak calculation. The first command allows reducing the mass shifts that occur during the spectra acquisition and excluding from the analysis all those spectra that do not satisfy the corresponding settings adjusted in spectra preparation parameters. After the recalibration step, a total average spectrum from the remaining individual spectra is calculated for each class. Thus, the software compared the generated average spectra for ATCC 842^T^, DSM 292, and DSM 365, and an average peak list table was created. The resulting peaks in such a list were used to retrieve characteristic peaks in the statistical analysis as well as for classification model generation. The analysis of characteristic peaks among ATCC 842^T^, DSM 292, and DSM 365 mass spectra was performed through three statistical approaches: multivariate unsupervised principal component analysis (PCA), *p*-value calculation in the average peak list, and supervised algorithms generation.

All the spectra were imported into the software to observe strain clustering by means of PCA analysis. The results of the PCA were visualised in the scores and loadings plots according to the first two components (PC1 and PC2), which explain most of the variance in the dataset (PC1 = 58% and PC2 = 20%).

Characteristic peaks among ATCC 842^ T^, DSM 292, and DSM 365 were selected and sorted through the following statistical tests: t-test, analysis of variance (ANOVA), the W/KW, and the AD test. The *p*-value cut-off was set at 0.05. First, the *p*-value of the AD test was calculated. More in detail, if the *p*-value in the AD test was > 0.05, the interesting peaks were selected among those having a *p*-value ≤ 0.05 in ANOVA analysis. Whereas, if the *p*-value in the AD test was ≤ 0.05, the selection was made among those having a *p*-value ≤ 0.05 in the W/KW^58^. Then, the characteristic peaks that best described the fingerprint of each strain were identified by the log base two (log_2_ times the change) of the ratio between the average peak intensities of the two strains under comparison (Table 1).

Moreover, Classification models were generated by ClinProTools software using the following algorithms: QC, SNN, and GA_KNN to obtain systematic and objective strain discrimination. In the GA, the following parameters were set: maximal number of peaks in the model (= 30), number of generations (= 50), and number of nearest neighbors in KNN classification (= 1 for KNN1; = 3 for KNN3; = 5 KNN5). In the QC algorithm, the sort/weight mode was set according to the *p*-value of W/KW. Each algorithm selects a list of peaks that are the most relevant in the separation of the strains. All models were validated using independent test data sets representing the classes. The external data sets for cross-validation were composed of 21 mass spectra replicates for ATCC 842^T^ and 20 for DSM 292 and DSM 365 selected from an independent analysis after assessing spectra quality by FlexAnalysis software (v 3.4; Bruker Daltonik GmbH, Bremen, Germany). According to the results, the model prediction capabilities were obtained by calculating the accuracy, sensitivity, and specificity.

The external data sets were also used to create the reference MSPs of DSM 292 and DSM 365 inside the *in-house* reference library. Subsequently, the identification results of the previously acquired fifteen spectra were reevaluated using the MBT Compass Explorer 4.100.1 module (Bruker Daltonik GmbH, Bremen, Germany). This reevaluation aimed to assess the impact of the library implementation with the new reference MSPs on the identification process of DSM 292 and DSM 365.

### 16S rRNA sequence analysis

The DNA was purified from an overnight culture of the strains ATCC 842^T^, DSM 292 and DSM 365 by means of the Wizard Genomic DNA purification Kit (Promega). The 16S rRNA gene was amplified with the primers E8F^59^ and E1541R^59^, and the PCR product was Sanger sequenced with the primers E27F^60^ and E1492R^60^. The electropherograms were analysed and assembled by means of BioNumerics 7.6, IUPAC degenerate nucleotide codes were inserted at uncertain peaks. The obtained sequence (GenBank Acc. No. OR506150, OR506151, OR506152) was compared in the EzBioCloud database. The most related species belonging to the *P. polymixa* group in the EzBiocloud database came from Sanger sequencing of PCR products (GenBank Acc. No. AF273740; AF391124; AJ320494; MH842737.1), except from three sequences retrieved from genomes: *P. polymyxa* ATCC 842^T^ (GenBank Acc. No: AFOX01000032:1540-65, on contig 32) and the only non type strain in the EzBioCloud *P. polymyxa* E681 (GenBank Acc. No CP000154: 2,525,414–2,526,891) and *Paenibacillus kribbensis* AM49 (GenBank Acc. No. CP020028: 618,273–619,838).

### Comparative genomics

The RefSeq assembly genome sequences GCF_903797665.1 (*P. polymyxa* ATCC 842^T^), GCF_900109125.1 (*P. polymyxa* DSM 292), and GCF_000714835.1 (*P. polymyxa* DSM 365) were used to calculate the ANI with the OrthoANI algorithm through the OAT software, which measures the overall similarity between two genomes without gene-finding and functional annotation steps^61^.

### Phenotypical test and fatty acids cellular composition profiles

Morphological characterisation, temperature optimum, salt tolerance, API gallery tests, and fatty acids cellular composition profiles were carried out by DSMZ Services, Leibniz Institut DSMZ—Deutsche Sammlung von Mikroorganismen und Zellkulturen GmbH, Braunschweig, Germany.

The fatty acids profile was analysed by gas chromatography of fatty methyl esters (GC-FAME), using minor modifications of the method of Miller^62^ and Kuykendall et al.^63^.

Biochemical characterisation, including the analysis of different substrate utilisation and enzyme production, was performed through API 50 CHB, API 20E, API 20NE, and API ZYM systems (Biomérieux) according to the manufacturer’s instructions.

### Supplementary Information

Supplementary Information.

## Data Availability

All data generated or analysed during this study are included in this article.

## References

[1] Patowary R, Deka H (2020). Beneficial Microbes in Agro-Ecology.

[2] Grady EN, MacDonald J, Liu L, Richman A, Yuan Z-C (2016). Current knowledge and perspectives of Paenibacillus: A review. Microb. Cell Fact..

[3] Kwak M-J (2020). Genome-based reclassification of *Paenibacillus jamilae* Aguilera et al.as a later heterotypic synonym of *Paenibacillus polymyxa* (Prazmowski 1880) Ash et al. Int. J. Syst. Evol. Microbiol..

[4] Ash C, Priest FG, Collins MD (1993). Molecular identification of rRNA group 3 bacilli (Ash, Farrow, Wallbanks and Collins) using a PCR probe test. Antonie Van Leeuwenhoek.

[5] Langendries S, Goormachtig S (2021). *Paenibacillus polymyxa*, a Jack of all trades. Environ. Microbiol..

[6] Lal S, Tabacchioni S (2009). Ecology and biotechnological potential of Paenibacillus polymyxa: A minireview. Indian J. Microbiol..

[7] Liu X, Li Q, Li Y, Guan G, Chen S (2019). Paenibacillus strains with nitrogen fixation and multiple beneficial properties for promoting plant growth. PeerJ.

[8] Coelho MRR, von der Weid I, Zahner V, Seldin L (2003). Characterization of nitrogen-fixing Paenibacillus species by polymerase chain reaction–restriction fragment length polymorphism analysis of part of genes encoding 16S rRNA and 23S rRNA and by multilocus enzyme electrophoresis. FEMS Microbiol. Lett..

[9] Lal S, Chiarini L, Tabacchioni S (2016). Bacilli and Agrobiotechnology.

[10] Raza, W., Yang, W. & Shen, Q. Paenibacillus polymyxa: antibiotics, hydrolytic enzymes and hazard assessment. *J. Plant Pathol.*, 419–430 (2008).

[11] Daud NS, Rosli MA, Azam ZM, Othman NZ, Sarmidi MR (2019). Paenibacillus polymyxa bioactive compounds for agricultural and biotechnological applications. Biocatal. Agric. Biotechnol..

[12] Tinôco D, Pateraki C, Koutinas AA, Freire DM (2021). Bioprocess Development for 2, 3-Butanediol Production by Paenibacillus Strains. ChemBioEng Rev..

[13] Dias BDC (2018). 2, 3-Butanediol production by the non-pathogenic bacterium Paenibacillus brasilensis. Appl. Microbiol. Biotechnol..

[14] Białkowska AM (2016). Strategies for efficient and economical 2, 3-butanediol production: New trends in this field. World J. Microbiol. Biotechnol..

[15] Xie N-Z (2015). Genome sequence of type strain *Paenibacillus polymyxa* DSM 365, a highly efficient producer of optically active (R, R)-2, 3-butanediol. J. Biotechnol..

[16] Kumar, S. & Ujor, V. C. Complete Genome Sequence of Paenibacillus polymyxa DSM 365, a Soil Bacterium of Agricultural and Industrial Importance. *Microbiol. Resour. Announce.*, e00329–00322 (2022). 10.1128/mra.00329-22 PMC9202436 35575559

[17] *Leibniz Institute DSMZ-German Collection of Microorganisms and Cell Cultures GmbH*, https://www.dsmz.de/collection/catalogue/details/culture/DSM-365 (2023).

[18] Park KY, Seo SY, Oh B-R, Seo J-W, Kim YJ (2018). 2, 3-Butanediol induces systemic acquired resistance in the plant immune response. J. Plant Biol..

[19] Heinze S (2020). Draft genome sequence of *Paenibacillus polymyxa* DSM 292, a gram-positive, spore-forming soil bacterium with high biotechnological potential. Microbiol. Resour. Announce..

[20] Heinze S (2018). Evaluation of promoter sequences for the secretory production of a Clostridium thermocellum cellulase in *Paenibacillus polymyxa*. Appl. Microbiol. Biotechnol..

[21] Weis CV, Jutzeler CR, Borgwardt K (2020). Machine learning for microbial identification and antimicrobial susceptibility testing on MALDI-TOF mass spectra: a systematic review. Clin. Microbiol. Infect..

[22] Holland JH (1992). Adaptation in Natural and Artificial Systems: An Introductory Analysis with Applications to Biology, Control, and Artificial Intelligence.

[23] Chun J (2018). Proposed minimal standards for the use of genome data for the taxonomy of prokaryotes. Int. J. Syst. Evol. Microbiol..

[24] Qadri S, Nichols C, Qadri S, Villarreal A (1978). Rapid test for acetyl-methyl-carbinol formation by enterobacteriaceae. J. Clin. Microbiol..

[25] Pérez-Sancho M (2018). Rapid differentiation of Staphylococcus aureus subspecies based on MALDI-TOF MS profiles. J. Vet. Diagn. Invest..

[26] Gekenidis M-T, Studer P, Wüthrich S, Brunisholz R, Drissner D (2014). Beyond the matrix-assisted laser desorption ionization (MALDI) biotyping workflow: In search of microorganism-specific tryptic peptides enabling discrimination of subspecies. Appl. Environ. Microbiol..

[27] Huang C-H, Huang L (2018). Rapid species-and subspecies-specific level classification and identification of Lactobacillus casei group members using MALDI Biotyper combined with ClinProTools. J. Dairy Sci..

[28] Suarez S (2013). Ribosomal proteins as biomarkers for bacterial identification by mass spectrometry in the clinical microbiology laboratory. J. Microbiol. Methods.

[29] Dematheis F (2022). Machine learning algorithms for classification of MALDI-TOF MS spectra from phylogenetically closely related species *Brucella melitensis*, *Brucella abortus* and *Brucella suis*. Microorganisms.

[30] Kann S (2020). MALDI-TOF mass spectrometry for sub-typing of Streptococcus pneumoniae. BMC Microbiol..

[31] Manzulli V (2021). Discrimination of Bacillus cereus group members by MALDI-TOF mass spectrometry. Microorganisms.

[32] Croxatto A, Prod'hom G, Greub G (2012). Applications of MALDI-TOF mass spectrometry in clinical diagnostic microbiology. FEMS Microbiol. Rev..

[33] Jeong H, Choi S-K, Ryu C-M, Park S-H (2019). Chronicle of a soil bacterium: Paenibacillus polymyxa E681 as a tiny guardian of plant and human health. Front. Microbiol..

[34] Celandroni F (2016). Identification and pathogenic potential of clinical Bacillus and Paenibacillus isolates. PLoS One.

[35] Kopcakova A (2022). The application of MALDI-TOF MS for a variability study of paenibacillus larvae. Vet. Sci..

[36] He Z (2007). Isolation and identification of a Paenibacillus polymyxa strain that coproduces a novel lantibiotic and polymyxin. Appl. Environ. Microbiol..

[37] Qi SS, Cnockaert M, Carlier A, Vandamme PA (2021). *Paenibacillus foliorum* sp. nov., *Paenibacillus phytohabitans* sp. nov., *Paenibacillus plantarum* sp. nov., *Paenibacillus planticolens* sp. nov., *Paenibacillus phytorum* sp. nov. and *Paenibacillus germinis* sp. nov., isolated from the Arabidopsis thaliana phyllosphere. Int. J. Syst. Evol. Microbiol..

[38] Tarfeen N, Nisa KU, Nisa Q (2022). MALDI-TOF MS: Application in diagnosis, dereplication, biomolecule profiling and microbial ecology. Proc. Indian Natl. Sci. Acad..

[39] Shu L-J, Yang Y-L (2017). Bacillus classification based on matrix-assisted laser desorption ionization time-of-flight mass spectrometry—Effects of culture conditions. Sci. Rep..

[40] Malviya D (2020). Lesson from ecotoxicity: Revisiting the microbial lipopeptides for the management of emerging diseases for crop protection. Int. J. Env. Res. Public Health.

[41] Jeong, H. *et al.* Draft genome sequence of the Paenibacillus polymyxa type strain (ATCC 842T), a plant growth-promoting bacterium. *J. Bacteriol.* **193** (2011). 10.1128/JB.05447-11 PMC3165700 21742878

[42] Vater J (2018). Genome mining of the lipopeptide biosynthesis of Paenibacillus polymyxa E681 in combination with mass spectrometry: Discovery of the lipoheptapeptide paenilipoheptin. ChemBioChem.

[43] Vater J, Niu B, Dietel K, Borriss R (2015). Characterization of novel fusaricidins produced by Paenibacillus polymyxa-M1 using MALDI-TOF mass spectrometry. J. Am. Soc. Mass Spectrom..

[44] Mülner P (2021). Fusaricidins, polymyxins and volatiles produced by *Paenibacillus polymyxa* strains DSM 32871 and M1. Pathogens.

[45] Ha M (2019). Reliable identification of Bacillus cereus group species using low mass biomarkers by MALDI-TOF MS. J. Microbiol. Biotechnol..

[46] Walczak-Skierska J, Monedeiro F, Maślak E, Złoch M (2023). Lipidomics characterization of the microbiome in people with diabetic foot infection using MALDI-TOF MS. Anal. Chem..

[47] Maślak E (2023). Silver nanoparticle targets fabricated using chemical vapor deposition method for differentiation of bacteria based on lipidomic profiles in laser desorption/ionization mass spectrometry. Antibiotics.

[48] Shu LJ, Yang YL (2017). Bacillus classification based on matrix-assisted laser desorption ionization time-of-flight mass spectrometry-effects of culture conditions. Sci. Rep..

[49] Ryzhov V, Fenselau C (2001). Characterization of the protein subset desorbed by MALDI from whole bacterial cells. Anal. Chem..

[50] Wieme AD (2014). Effects of growth medium on matrix-assisted laser desorption-ionization time of flight mass spectra: A case study of acetic acid bacteria. Appl. Environ. Microbiol..

[51] Vishnoi A, Roy R, Bhattacharya A (2007). Comparative analysis of bacterial genomes: Identification of divergent regions in mycobacterial strains using an anchor-based approach. Nucleic Acids Res..

[52] Zhou Y, Zhang W, Wu H, Huang K, Jin J (2019). A high-resolution genomic composition-based method with the ability to distinguish similar bacterial organisms. BMC Genom..

[53] Choo, S. W., Rishik, S. & Wee, W. Y. Comparative genome analyses of Mycobacteroides immunogenum reveals two potential novel subspecies. *Microb. Genom.* **6** (2020). 10.1099/mgen.0.000495 PMC8116688 33295861

[54] Maasz G (2020). Testing the applicability of MALDI-TOF MS as an alternative stock identification method in a cryptic species complex. Molecules.

[55] Rahi P, Prakash O, Shouche YS (2016). Matrix-assisted laser desorption/ionization time-of-flight mass-spectrometry (MALDI-TOF MS) based microbial identifications: Challenges and scopes for microbial ecologists. Front. Microbiol..

[56] Wilson DA (2017). Multicenter evaluation of the bruker MALDI biotyper CA system for the identification of clinically important bacteria and yeasts. Am. J. Clin. Pathol..

[57] Elssner T, Kostrzewa M (2006). CLINPROT-a MALDI-TOF MS based system for biomarker discovery and analysis. Clin. Proteom..

[58] Stephens MA (1974). EDF statistics for goodness of fit and some comparisons. J. Am. Stat. Assoc..

[59] Baker G, Smith JJ, Cowan DA (2003). Review and re-analysis of domain-specific 16S primers. J. Microbiol. Methods.

[60] Soergel DA, Dey N, Knight R, Brenner SE (2012). Selection of primers for optimal taxonomic classification of environmental 16S rRNA gene sequences. ISME J..

[61] Lee I, Kim YO, Park S-C, Chun J (2016). OrthoANI: An improved algorithm and software for calculating average nucleotide identity. Int. J. Syst. Evol. Microbiol..

[62] Miller LT (1982). Single derivatization method for routine analysis of bacterial whole-cell fatty acid methyl esters, including hydroxy acids. J. Clin. Microbiol..

[63] Kuykendall L, Roy M, O’neill J, Devine T (1988). Fatty acids, antibiotic resistance, and deoxyribonucleic acid homology groups of Bradyrhizobium japonicum. Int. J. Syst. Evol. Microbiol..

