# Supplementary material for: MALDI-TOF as a powerful tool for identifying and differentiating closely related microorganisms: the strange case of three reference strains of Paenibacillus polymyxa
Source: Sci Rep. 2024 Jan 31;14:2585. doi: 10.1038/s41598-023-50010-w (PMC10831075; doi:10.1038/s41598-023-50010-w)
Supplement: Supplementary file 1 — Supplementary Information. [file 41598_2023_50010_MOESM1_ESM.docx]

**Supplementary information**

**MALDI-TOF as a powerful tool for identifying and differentiating closely related microorganisms: the strange case of three reference strains of *Paenibacillus polymyxa***

Ilaria Lebano

Fabio Fracchetti

Mario Li Vigni,

Juan Fernando Mejia

Giovanna Felis

Silvia Lampis

| **Table of contents** | | |
| --- | --- | --- |
| **Figure S1** | ***Paenibacillus polymyxa* ATCC 842^T^, DSM 292 and DSM 365 cells observation (vegetative cells and endospores) under microscope** | 3 |
| **Figure S2** | **Three- and two-dimensional principal component analysis and respective loadings plots of P. polymyxa ATCC 842^T^, DSM 292 and DSM 365 strains.** | 4 |
| **Figure S3** | **Average spectra view of P. polymyxa ATCC 842^T^, DSM 292 and DSM 365 generated through ClinProTools Software.** | 5 |
| **Table S1** | **Morphological and biochemical characterization of ATCC 842^T^, DSM 292 and DSM 365.** | 6 |
| **Table S2** | **Results of API 20E system (Biomérieux) recorded after 48 hours post-incubation at 28°C.** | 7 |
| **Table S3** | **Results of API 50 CHB system (Biomérieux) recorded after 48 hours post*-*incubation at 28°C.** | 8-9 |
| **Table S4** | **Results of API 20 NE system (Biomérieux) recorded after 48 hours post-incubation at 28°C.** | 10 |
| **Table S5** | **Results of API ZYM system (*Biomérieux*) recorded after 6 hours *post-incubation* at 37°C.** | 11 |


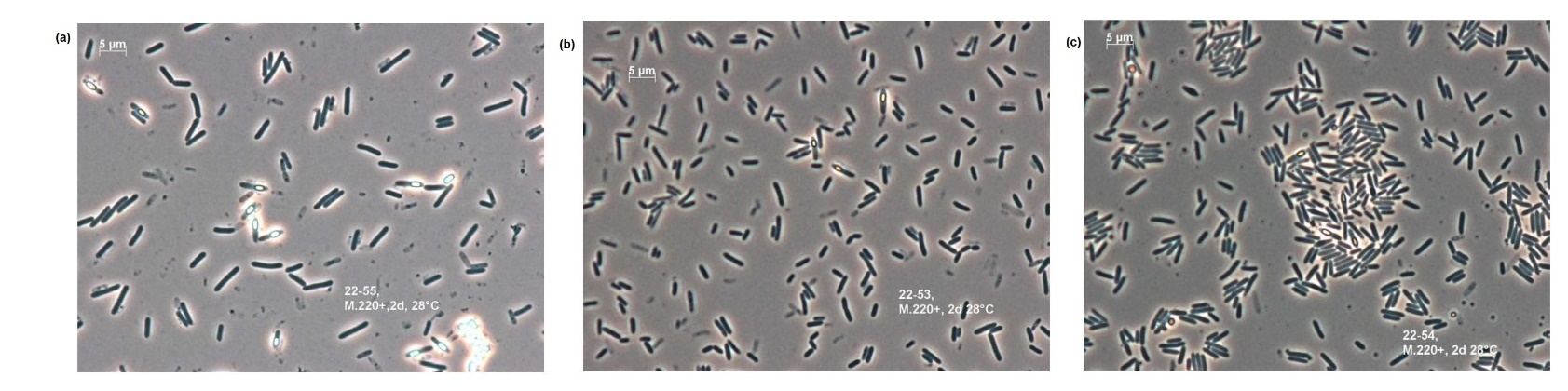


**Figure S1. Vegetative cells and endospore observation of (a) ATCC 842T, (b) DSM 292 and (c) DSM 365.** The strains were grown at 28°C on M220 medium supplemented with MnSO4 for 48 hours. Samples were examinated by Axio Scope.A1 microscope (Zeiss) with objective oil len 100X. Pictures were made with Axio Cam MRc Zeiss using the Axiovision Rel 4.8. software. Scale bars represent 5 µm.


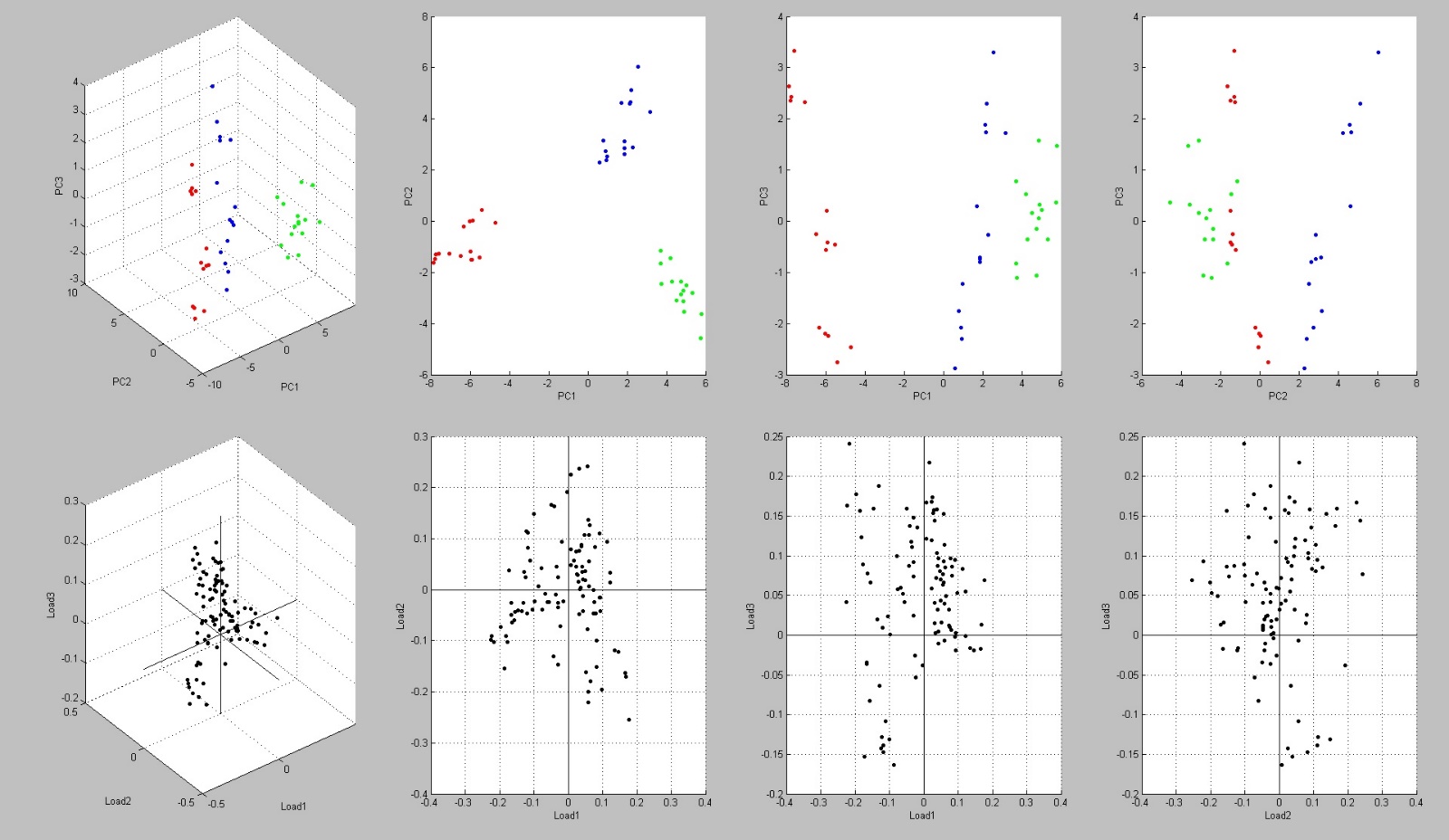


**Figure S2. Three- and two-dimensional principal component analysis and respective loadings plots of P. polymyxa ATCC 842^T^ (red), DSM 292 (green), and DSM 365 (blue) strains.**


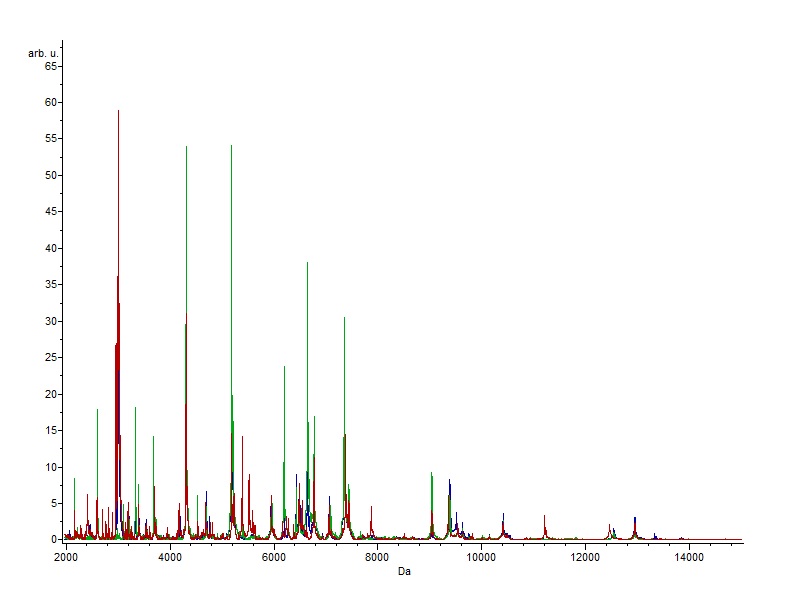


**Figure S3. Average spectra view of P. polymyxa ATCC 842^T^ (red), DSM 292 (green) and DSM 365 (blue) generated through ClinProTools Software** (v 3.0; Bruker Daltonics, Bremen, Germany) and processed following the standard data preparation workflow (baseline subtraction, normalisation, recalibration, average peak list calculation, and peak calculation).

**Table S1. Morphological and biochemical characterization of ATCC 842^T^, DSM 292 and DSM 365.**

| **Feature** | **ATCC 842^T^** | **DSM 292** | **DSM 365** |
| --- | --- | --- | --- |
| Cell Form | rods | rods | rods |
| Cell Size | 4-11 x 0.8 | 1-3 x 0.8 | 2-3 x 0.8 |
| Gram Behaviour | + | + | + |
| Catalase | + | + | + |
| Oxidase | - | - | - |
| Sporulation | + | + | + |
| Sporangium Swollen | + | + | + |
| Motility | + | + | + |
| Anaerobic Growth | + | + | + |
| Gas Production | + | + | + |
| 5% NaCl | w | - | w |
| 8% NaCl | - | - | - |
| PH 5 | + | + | + |
| PH 7 | + | + | + |
| PH 11 | - | - | - |
| 5°C | - | - | - |
| 25°C | + | + | + |
| 30°C | + | + | + |
| 35°C | w | w | + |
| 40°C | (-) | (-) | (-) |
| Tween 20 | + | + | + |
| Tween 40 | + | + | + |
| Tween 80 | w | - | + |
| Casein | + | + | + |

w = weak; (-) = very weak; += positive

**Table S2: Results of API 20E system (Biomérieux) recorded after 48 hours post-incubation at 28°C.**

| **Feature** | **ATCC 842^T^** | **DSM 292** | **DSM 365** |
| --- | --- | --- | --- |
| β-Galactosidase (ONPG) | + | + | + |
| Arginine dihydrolase (ADH) | - | - | - |
| Lysine decarboxylase (LDC) | - | - | - |
| Ornithine decarboxylase (ODC) | - | - | - |
| Citrate utilization | - | - | - |
| H2S production | - | - | - |
| Urease | - | - | - |
| Tryptophan deaminase | - | - | - |
| Indole | - | - | - |
| Voges-Proskauer (VP) | w | + | + |
| Gelatine hydrolysis | + | + | + |
| Acid from |  |  |  |
| Glucose | - | - | - |
| Mannose | - | - | - |
| Inositol | - | - | - |
| Sorbitol | - | - | - |
| Rhamnose | - | - | - |
| Sucrose | - | - | - |
| Melibiose | - | - | - |
| Amygdalin | - | - | - |
| Arabinose | - | - | - |

w = weak; - = negative; + = positive

**Table S3: Results of API 50 CHB system (Biomérieux) recorded after 48 hours post-incubation at 28°C.**

| **Feature** | **ATCC 842^T^** | **DSM 292** | **DSM 365** |
| --- | --- | --- | --- |
| Glycerol | + | + | + |
| Erythritol | - | - | - |
| D-Arabinose | - | - | - |
| L-Arabinose | + | + | + |
| Ribose | + | + | + |
| D-Xylose | + | + | + |
| L-Xylose | - | - | - |
| Adonitol | - | - | - |
| βMeDXyloside | + | + | + |
| Galactose | + | + | + |
| Glucose | + | + | + |
| Fructose | + | + | + |
| Mannose | + | + | + |
| Sorbose | - | - | - |
| Rhamnose | - | + | - |
| Dulcitol | - | - | - |
| Inositol | - | - | - |
| Mannitol | + | + | + |
| Sorbitol | - | - | - |
| αMeDMannoside | - | + | - |
| αMeGlucoside | + | + | + |
| N-Acetylglucosamine | - | ? | - |
| Amygdalin | + | + | + |
| Arbutin | + | + | + |
| Esculin | + | + | + |
| Salicin | + | + | + |
| Cellobiose | + | + | + |
| Maltose | + | + | + |
| Lactose | + | + | + |
| Melibiose | + | + | + |
| Sucrose | + | + | + |
| Trehalose | + | + | + |
| Inulin | ? | + | + |
| Melizitose | - | - | - |
| Raffinose | + | + | + |
| Starch | + | + | + |
| Glycogen | + | + | + |
| Xylitol | - | - | - |
| Gentibiose | + | + | + |
| D-Turanose | + | + | + |
| D-Lyxose | - | - | - |
| D-Tagatose | - | - | - |
| D-Fucose | - | - | - |
| L-Fucose | - | - | - |
| D-Arabitol | - | - | - |
| L-Arabitol | - | - | - |
| Gluconate | ? | ? | ? |
| 2Ketogluconate | - | - | - |
| 5Ketogluconate | - | - | - |

? = no clear result; - = negative; + = positive

**Table S4: Results of API 20 NE system (Biomérieux) recorded after 48 hours post-incubation at 28°C.**

| **Feature** | **ATCC 842^T^** | **DSM 292** | **DSM 365** |
| --- | --- | --- | --- |
| Nitrate reduction | + | + | + |
| Indole | - | - | - |
| Glucose fermentation | - | - | - |
| Arginine dihydrolase | - | - | - |
| Urease | - | - | - |
| Esculin hydrolysis | + | + | + |
| Gelatine hydrolysis | + | + | + |
| β-Galactosidase | + | + | + |
| Glucose assimilation | + | + | + |
| Arabinose assimilation | + | + | + |
| Mannose assimilation | + | + | + |
| Mannitol assimilation | + | + | + |
| N-Acetylglucosamine assimilation | - | - | - |
| Maltose assimilation | + | + | + |
| Gluconate assimilation | + | + | + |
| Caprate assimilation | - | - | - |
| Adipate assimilation | - | - | - |
| Malate assimilation | - | + | - |
| Citrate assimilation | - | - | - |
| Phenylacetate assimilation | - | - | - |

- = negative; + = positive

**Table S5: Results of API ZYM system (Biomérieux) recorded after 6 hours post-incubation at 37°C.**

| **Feature** | **ATCC 842^T^** | **DSM 292** | **DSM 365** |
| --- | --- | --- | --- |
| Alcaline Phosphatase | 0.5 | 0.5 | 1 |
| Esterase | 3 | 3 | 3 |
| Esterase Lipase | 1 | 1 | 1 |
| Lipase | 0 | 0 | 0 |
| Leucine-Arylamidase | 0 | 1 | 0 |
| Valine-Arylamidase | 0.5 | 0.5 | 0.5 |
| Cystine-Arylamidase | 0 | 0 | 0 |
| Trypsin | 0 | 0 | 0 |
| Chymotrypsin | 0.5 | 0 | 0 |
| Acid Phosphatase | 0.5 | 0 | 0 |
| Naphtol-AS-BI-Phosphohydrolase | 0.5 | 1 | 0.5 |
| α-Galactosidase | 1 | 1 | 2 |
| β-Galactosidase | 3 | 4 | 5 |
| β-Glucuronidase | 0 | 0 | 0 |
| α-Glucosidase | 0 | 0 | 0 |
| β-Glucosidase | 1 | 0.5 | 0.5 |
| N-Acetyl-β-Glucosaminidase | 0 | 0 | 0 |
| α-Mannosidase | 0 | 0 | 0 |
| α-Fucosidase | 0 | 0 | 0 |
